# The impact of a workplace virtual reality-delivered exercise program on central hemodynamics in people with developmental disability living in Australia: A pilot randomised waitlist-controlled study

**DOI:** 10.1016/j.pmedr.2025.103256

**Published:** 2025-09-29

**Authors:** Joyce S. Ramos, June Alexander, Lance C. Dalleck, Claire Drummond, Alline Beleigoli, Belinda Lange, Caroline Ellison, On behalf of the Cardiometabolic, Neuro Rehabilitation Group

**Affiliations:** a Flinders University, Caring Futures Institute, College of Nursing and Health Sciences, Australia; b University of South Australia, Ageing and Disability, Australia; c Western Colorado University, Recreation, Exercise & Sport Science Department, United States; d Flinders University, College of Science and Engineering, Medical Device Research Institute, Australia; e Flinders University, College of Medicine and Public Health, Rural and Remote Health SA, Australia

**Keywords:** Developmental disability, Virtual reality, Exercise, Cardiovascular health, Central hemodynamics

## Abstract

**Objectives:**

Abnormalities of central hemodynamic indices (CHI) elevate risk of cardiovascular events (CVE) and all-cause mortality. Most people with developmental disability do not meet the exercise recommendations necessary to improve health outcomes, including CHI. The aim of this pilot study was to investigate the impact of a workplace integrated virtual reality-delivered exercise (VR-E) program compared with no intervention on CHI in people with developmental disability.

**Methods:**

Seventeen people with developmental disability employed at a disability service were randomised into VR-E (*n* = 8) or waitlist-control (*n* = 9). The VR-E group completed a 1-h supervised session at the workplace, three times/week for eight weeks. Following an eight-week period, the waitlist-control group also underwent the program. CHI were assessed at pre- and post-intervention via cuff oscillometry.

**Results:**

There are 21 complete CHI data (VR-E, *n* = 13; waitlist-control, *n* = 8) from pre- to post-8-week period. There were no significant between-group differences in CHI changes from pre- to post-8-week period (*p* > 0.05). However, although not statistically significant between-groups (augmentation index at 75 beats per minute [AIx75], *p* = 0.7; forward pressure wave [Pfw], *p* = 0.6; backward pressure wave [Pbw], p = 0.7), the VR-E group showed a small improvement in CHI including AIx75 (−4 ± 11 %, d = 0.23), Pfw (−1.5 ± 8 mmHg,d = 0.18), and Pbw (−0.7 ± 5 mmHg,d = 0.25). Whereas the waitlist-control group showed negligible changes in these CHI (AIx75, −1 ± 19 %, d = 0.06; Pf, 0.5 ± 4 mmHg, d < 0.01; Pb, <0.01 ± 4 mmHg, d < 0.01) from pre- to post-study.

**Conclusions:**

This pilot study suggests that a workplace-integrated VR-E may be a viable intervention to improve CHI, which may indicate reduced CVE risk and all-cause mortality in people with developmental disability.

## Highlights

• Integrating exercise programs in a workplace using virtual reality may be safe.
• A workplace-integrated virtual reality exercise program could enhance heart health.
• Implementing a workplace virtual reality exercise may improve functional independence.

## 1 Introduction

Developmental disabilities refer to conditions that occurred as an infant or during foetus development, resulting in physical, learning, language, and behavioural impairments. These include conditions such as intellectual disability, autism spectrum disorder, attention deficit, learning disability, and down syndrome (Zablotsky et al., 2019). This population experience health disparities including increased occurrence of adverse health conditions, poor access to quality health care services, and insufficient emphasis on health promotion, relative to the general population (Krahn et al., 2006). Management strategies such as nutrition, physical activity/exercise, and social interventions have therefore been implemented for the health promotion and disease prevention of people with developmental disability (Carmeli and Imam, 2014). Specifically, there has been an emphasis on improving physical activity/exercise, given that inactivity has been shown to be a major contributor to ill health in this population (Jacob et al., 2023). Most individuals with developmental disability do not meet the global exercise recommendations for optimal cardiovascular health (Rozak et al., 2017), significantly increasing their risk of cardiovascular events (CVE) such as a heart attack or stroke (Erickson et al., 2016).

Central hemodynamic indices (CHI) are well established independent predictors of CVE (Chirinos et al., 2012) and all-cause mortality (Li et al., 2019). These are parameters that reflect the flow and pressure that the heart is required to generate and efficiently supply oxygenated blood throughout the cardiovascular system (Pappano and Wier, 2013). There is evidence to suggest that people with developmental disability have less favourable CHI relative to their healthy counterparts (Hilgenkamp et al., 2019). Our research team has also previously shown that increased cardiorespiratory fitness with exercise is associated with favourable CHI, specifically in individuals with increased risk of CVE (Ramos et al., 2016). It is therefore of significant importance to promote regular exercise adherence in those with developmental disability.

The tendency of those with developmental disability to avoid regular exercise has been attributed to low motivation and reduced access to mainstream health and fitness services (MacDonald et al., 2022). There is therefore a need to implement programs that incorporate motivational factors and those that can be delivered remotely or integrated within a workplace environment. A growing body of evidence support the efficacy of digital health tools as a self-management strategy and caregiver engagement to promote long-term care for people with disabilities (Vázquez et al., 2018), particularly in those located in remote or rural areas with limited access to mainstream health care (Gallego et al., 2017). Specifically, the use of virtual reality to promote exercise has been shown to improve physical fitness of individuals with a range of developmental disability (Lotan et al., 2010). Virtual reality-delivered exercise (VR-E) programs conducted for five to12 weeks have been reported to motivate this population enough to participate in exercise activities and induce physical fitness improvement (Lotan et al., 2010; Li et al., 2023). However, the specific virtual reality systems used varied between these studies or are now considered obsolete or expensive (DuBose, 2022).

In recent years, the cost of commercially available virtual reality systems has significantly plummeted. Additionally, contemporary virtual reality systems are now able to offer a variety of safe activities that may be individually tailored to the interests, needs, or goals of persons with disabilities, specifically activities that were traditionally inaccessible. For example, individuals who require mobility aids can engage in virtual reality-delivered sports activities within a safe environment that they could not otherwise experience. Despite this potential, methodological differences and insufficient emphasis on clinical outcomes like CHI alongside physical fitness metrics in previous studies leave the cardiovascular health benefits of VR-E programs uncertain in people with developmental disabilities.

The aim of this pilot study was to investigate the impact of a workplace integrated VR-E program compared with no intervention on CHI in people with developmental disability. It was hypothesised that a workplace integrated VR-E program would improve CHI more than no intervention in this population. Demonstrating the efficacy of a VR-E program provides critical research evidence to support the integration of technology in clinical practice for people with developmental disabilities, thereby empowering clinicians and caregivers with more strategies to encourage exercise in this population.

## 2 Methods

**Fig. 1:**
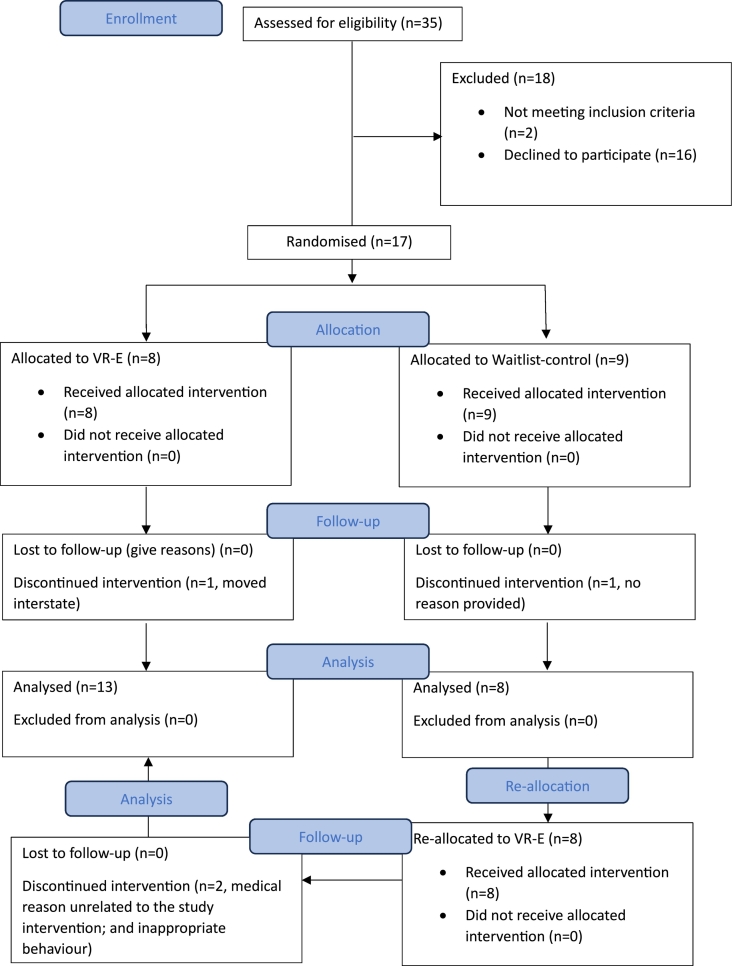
Consort flow diagram of the pilot study conducted at Bedford Group disability service in Australia in 2023; VR-E = virtual reality-based exercise group.

Seventeen participants with developmental disability and working at a disability employment service (Bedford Group) were randomised (stratified by age and sex) into either the VR-E (*n* = 8) or waitlist-control (*n* = 9) group (Fig. 1). The VR-E group completed a one- hour supervised session within the workplace, three times per week for eight weeks. Following an eight-week period, the waitlist-control group also underwent the VR-E program, which adheres to ethical research principles by ensuring all participants eventually have the opportunity to benefit from the potential benefits of the intervention under investigation. The sample size was based on the number of participants from Bedford Group who agreed to be included during the study recruitment period (January 2023 to March 2023). The randomisation and enrolment procedure were performed by a blinded investigator using a software employing random permuted blocks. The blinded investigator is not included in the application of the intervention. Recruitment was conducted by contacting the Bedford Group staff to explain the study and to provide the eligibility criteria and written information for participants. Bedford Group staff then identified potential participants and provided information about the study. A Flinders researcher with expertise in employment outcomes for people with developmental disability met with potential participants and provided confirmation of the potential participants' interest and understanding of the study and further information if required. Flinders researchers confirmed the eligibility of all participants. Written and oral consent were obtained by an experienced researcher in the disability field prior to inclusion. Potential participants were also encouraged to have an advocate present at the time of confirming their participation. Participants were excluded if they presented with any of the following: diagnosed with epilepsy or recent history of seizures, limited arm and hand movement that would make it difficult to interact with VR technology, visual impairments, unstable angina, severe valvular heart disease, pulmonary disease, cardiomyopathy, recent myocardial infarction (last four weeks), uncontrolled hypertension, and kidney failure. This study was approved by the Flinders University Human Ethics Research Committee (approval number: 4524).

The participants underwent several tests in two-hours at their workplace (Bedford Group, Panorama, Adelaide, South Australia, Australia) to assess all primary (CHI) and secondary (muscular strength and mobility) outcome measures at baseline and eight-week follow-up. Participants were instructed by research personnel in the disability field to refrain from strenuous activities for at least 48 h, alcohol and caffeine for at least 24 h, and fast for eight hours before each assessment. The assessments were administered at approximately the same time of the day (morning, ± two hours) by an assessor blinded to the study allocation.

### 2.1 Virtual reality-delivered exercise program

**Fig. 2:**
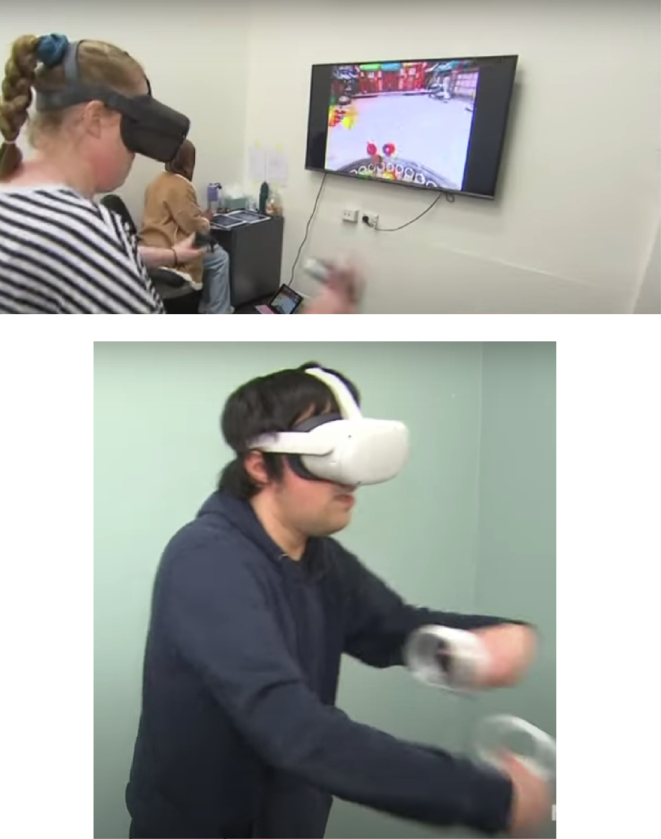
Schematic representation of VR-E program conducted at Bedford Group disability service in Australia in 2023.

Participants completed a one-hour supervised session (Fig. 2), three times per week, for eight weeks at the workplace (disability service). The intervention adherence was measured as the percentage of prescribed session attended by the participants. The rate of perceived exertion (RPE) during each of the VR-E session was monitored via the Borg 0–10 RPE scale (Williams, 2017) to determine the exercise intensity. Each session consisted of i) 10-min virtual reality set-up and selection of available virtual reality games; and ii) 50-mins of exercise using one of the following applications depending on the participant preference and goals: Dance Central (Harmonix), Beat Saber (Beat games), The Thrill of the Fight (Sealost Interactive), Space Pirate Trainer (I-Illusions), Fruit Ninja (Halfbrick Studios), OhShape (Odders lab), Racket NX (One Hamsa), Racket Fury: Table Tennis VR (Pixel Edge Games), Racket Fury, Swords of Gargantua (Thirdverse Yomuneco Inc), BoxVR (FitXR), Superhot VR (Superhot), VZFit Play, and VZFit Explorer (VirZoom); QuiVr (Barefoot Gaming); Premium Bowling (Sadetta); Real VR Fishing (Devs United Games); Everybody's Golf VR (Clap Hanz); The Climb (Crytek); VR basketball (IRL Studios).

### 2.2 Central hemodynamic indices

The CHI assessments were conducted in a supine position and quiet dimly lit room, following an eight-hour overnight fast. Before the tests, participants were required to rest for 10 min. All assessments were conducted using the cuff oscillometric method for pulse wave analysis (Wassertheurer et al., 2010) via the SphygmoCor Xcel cuff device (AtCor Medica, Sydney, Australia). This non-invasive and relatively operator-independent method has been shown to be comparable to radial tonometry for the estimation of CHI and has good reproducibility (Climie et al., 2012). An appropriate cuff size of the SphygmoCor Xcel device was fitted on the participants' upper arm once ready in a supine position. A blood pressure measurement with the cuff was first obtained via an automatic recording of standard oscillometric brachial blood pressure. This was then immediately followed by an automatic re-inflation of the cuff to a sub-diastolic pressure level which was held for a period of five *sec*. During this five-sec period, volumetric waveforms were recorded and subsequently calibrated against the brachial-cuff measured systolic and diastolic blood pressure prior to the application of a generalised transfer function to estimate a central pressure waveform. The central pressure waveform was then used to calculate different CHI. Augmentation index (AIx) was calculated as the difference between the first (Peak_1_) and the second (Peak_2_) systolic peaks, presented as a percentage of the central pulse pressure (cPP) waveform (100 × (Peak₂ - Peak₁) / cPP), where cPP was calculated as the difference between central systolic and diastolic blood pressure. AIx was also standardised to a heart rate of 75 beats per min (AIx75) as it is known to be a marker of left ventricular systolic loading that is dependent on heart rate. The SphygmoCor Cardiovascular Management System software (version 9, AtCor Medical) also automatically applied a wave separation analysis to obtain forward (Pfw) and backward (Pbw) pressure waveforms according to the wave reflection theory (Hametner et al., 2014). Reflection magnitude was also determined as the ratio of Pbw to Pfw, expressed as a percentage (Reflection Magnitude = (Pbw/Pfw) x 100).

### 2.3 Muscular strength and mobility

Objective measures of muscular strength and mobility were administered in the following order at each study timepoint: 30 s sit-to-stand (30 s STS) test, 30 s bicep curl test, and timed up and go (TUG) test.

#### 2.3.1 30s sit-to-stand

The 30 s STS was used to assess lower body muscle strength. This test required participants to repeatedly stand and sit down as fast and as safely as possible within 30 s from a standard chair placed against a wall. One repetition was counted as the successful completion of a cycle of standing up and sitting down from a chair. The number of completed repetitions (reps) within 30 s was recorded (Marlow et al., 2014).

#### 2.3.2 30 s bicep curl test

Upper body strength was assessed using the 30 s bicep curl test. This test was conducted in a seated position, with the feet flat on the floor and the back in an upright position. A two kg (females) or three kg (males) dumbbell was held in a neutral handshake grip position with the arm hanging on the side of the body before the test. Participants were required to flex at the elbow whilst supinating the forearm and to return to the initial position as fast and as safely as possible. The elbow must be positioned against the trunk throughout the duration of the test. The number of completed reps within 30 s was recorded for both arms (Marlow et al., 2014).

#### 2.3.3 Timed up and go test

Mobility was assessed using the TUG test which measured the participant's ability to rise from a chair, walk towards a brightly coloured cone that is located three metres in front of the chair, and return to a seated position in the chair. The participants were allowed two attempts to complete the test, with 30 s between trials. The best trial time to complete the test was recorded (Coombes and Skinner, 2020).

### 2.4 Statistical analysis

A per-protocol analysis was used. The Shapiro-Wilk test was used to test for assumptions of normality. If the assumption of normality was violated even after data log transformation, a non-parametric test equivalent was used. A two-way analysis of covariance was used to determine whether there is a significant difference in the change in outcome measures from pre- to post-intervention between groups (VR-E vs waitlist-control), wherein the difference in change value was deemed as the dependent factor and the baseline value as the covariate. The homogeneity of variances was assessed using the Levene's test. The Eta squared (η^2^) group × time interaction effect sizes were calculated as the between-group sum of squares divided by the total sum of squares and interpreted as follows: ‘small’ effect (0.01); ‘small-to-medium’ effect (0.01–0.10); ‘medium to-large’ (0.10–0.25). Within-group effect sizes (Cohen's d) were also calculated and interpreted as follows: small (d = 0.2), medium (d = 0.5), large (d = 0.8) (Vacha-Haase and Thompson, 2004). Significance level was set at *p* < 0.05. Data were analysed using the SPSS version 28 software package (IBM, New York, NY, USA).

## 3 Results

Of the 17 participants originally randomised, four participants discontinued the study, resulting to a total of 21 (VR-E, *n* = 13; waitlist-control, *n* = 8) complete pre- and post-data for the primary outcome of the pilot study (Fig. 1). Participants completed 91.7 % of the prescribed VR-E sessions, with the average exercise intensity reported to be in the RPE of four (‘somewhat hard’). There were no reported physical injuries that were directly related to the prescribed study intervention. Table 1 presents the participants' characteristics at baseline. The age, sex, and height of participants were similar between groups. The VR-E group had higher body mass, body mass index, and waist circumference relative to the waitlist-control group at baseline. On average, these anthropometric measures classified both study groups as overweight or obese, at increased risk of chronic disease (Government, 2025).

**Table 1:** Descriptive characteristics of adults with developmental disability who completed the study conducted at the Bedford Group disability service in Australia in 2023.

Variables	VR-E (*n* = 13)	Waitlist-control (*n* = 8)
Age, years	36 ± 12	36 ± 13
Male sex, %	77	67
Body mass, kg	90 ± 22	81 ± 19
Height, m	1.7 ± 0.1	1.7 ± 0.1
Body mass index, kg/m^2^	31 ± 10	29 ± 8
Waist circumference, cm	96 ± 14	90 ± 12

Data are presented as mean ± SD, VR-E, virtual reality-based exercise group.

### 3.1 Central hemodynamic indices

Table 2 presents the changes in CHI from pre to post study period between groups. There were no significant between-group differences in CHI changes from pre- to post-eight-week period (*p* > 0.05). However, although not statistically significant between group (AIx75, *p* = 0.7); Pfw, *p* = 0.6; Pbw, p = 0.7), the VR-E group showed a small improvement in CHI including AIx75 (−4 ± 11 % [CI -13 to 4 %], Cohen's d = 0.23), Pfw (−1.5 ± 8 mmHg [CI -5 to 2 mmHg], Cohen's d = 0.18), Pbw (−0.7 ± 5 mmHg [CI -3 to 2 mmHg], Cohen's d = 0.25). Whereas the waitlist-control group showed negligible changes in these CHI (AIx75, −1 ± 19 % [−12 to 10 %], Cohen's d = 0.06; Pfw, 0.5 ± 4 mmHg [−4 to 4 mmHg], Cohen's d < 0.01; Pbw, <0.01 ± 4 mmHg [−3 to 3 mmHg], Cohen's d < 0.01) from pre to post study period.

**Table 2:** Change in central hemodynamics, exercise capacity and muscular strength outcomes from pre- to post-8-week study period in adults with developmental disability who completed the study conducted at the Bedford Group disability service in Australia in 2023.

	VR-E (n = 13)			Effect size within group	Waitlist-control (n = 8)			Effect size within group	Group difference	
	Baseline	Post	Average Difference (95 % CI)	Cohen's d or r (magnitude)	Baseline	Post	Average Difference (95 % CI)	Cohen's d or r (effect size)	*P*-value adjusted for baseline value	Eta squared adjusted for baseline value (magnitude)
*Central hemodynamics*										
AIx75, %	14 ± 15	10 ± 19	−4 ± 11 (−13 to 4)	0.23 (small)	17 ± 12	16 ± 21	-1 ± 19 (−12 to 10)	0.06 (negligible)	0.7	0.01 (small)
AIx, %	16 ± 12	12 ± 18	-4 ± 11 (−12 to 4)	0.26 (small)	19 ± 11	19 ± 20	−0.3 ± 17 (−10 to 9)	<0.01 (negligible)	0.6	0.02 (small)
Augmented pressure, mm Hg	5 ± 4	5 ± 7	−0.8 ± 6 (−5 to 3)	<0.01 (negligible)	6 ± 4	6 ± 7	−0.5 ± 6 (−5 to 4)	<0.01 (negligible)	0.8	<0.01 (negligible)
Pfw, mm Hg	29 ± 4	28 ± 7	−1.5 ± 8 (−5 to 2)	0.18 (small)	29 ± 3	29 ± 3	0.5 ± 4 (−4 to 4)	<0.01 (negligible)	0.6	0.01 (small)
Pbw, mm Hg	15 ± 4	14 ± 4	−0.7 ± 5 (−3 to 2)	0.25 (small)	15 ± 3	15 ± 3	<0.01 ± 4 (−3 to 3)	<0.01 (negligible)	0.7	0.01 (small)
Reflection magnitude, %	49 ± 8	50 ± 10	1 ± 6 (−6 to 7)	0.11 (small)	53 ± 8	54 ± 16	1 ± 17 (−7 to 11)	0.08 (negligible)	0.8	<0.01 (negligible)
*Mobility and muscular strength*										
TUG, sec	8.5 ± 1.9	7.9 ± 1.6	−0.6 ± 2 (−1.8 to 0.5)	0.34 (small)	8.7 ± 1.9	8.9 ± 2.3	0.2 ± 1.8 (−1.3 to 1.6)	0.09 (negligible)	0.3	0.06 (medium)
Right 30s Bicep curls, reps	12 ± 3	15 ± 3	3 ± 2 (1 to 4)	1.0 (large)	11 ± 4	12 ± 3	2 ± 3 (−1 to 4)	0.28 (small)	0.1	0.10 (medium)
Left 30s Bicep curls, reps	13 ± 3	15 ± 4	2 ± 3 (0.5 to 4)	0.57 (medium)	11 ± 5	13 ± 3	2 ± 4 (−1 to 5)	0.49 (medium)	0.4	0.03 (small)
30s STS, reps	11 ± 3	11 ± 3	0 ± 2 (−1 to 1)	<0.01 (negligible)	9 ± 2	11 ± 3	1 ± 3 (−1 to 4)	0.78 (large)	0.5	0.02 (small)

Data are presented as mean ± SD; AIx75, augmentation index at 75 beats per minute; AIx, augmentation index; Pfw, forward pressure wave; Pbw, backward pressure wave; VR-E, virtual reality-based exercise program; TUG, Timed Up and Go; 30s STS; 30s sit-to-stand; CI, confidence interval; p-value, derived from two-way analysis of covariance.

### 3.2 Muscular strength and mobility

Table 2 shows the changes in muscular strength, and mobility from baseline to post-study period between groups. There were no significant between-group differences (*p* > 0.05) in muscular strength (30 s STS, *p* = 0.51; and 30 s bicep curls, *p* = 0.11) and mobility (TUG, *p* = 0.25). Although not statistically significant, there was a large increase in right-arm muscle strength as reflected by the increased number of bicep curl reps following VR-E (+3 ± 2 reps [CI 1 to 4 reps], Cohen's d = 1.0) compared to only a small improvement following waitlist-control (+2 ± 3 reps [−1 to 4 reps], Cohen's d = 0.28). There was also a small improvement in mobility as reflected by a decreased time to complete the TUG test following VR-E (−0.6 ± 2 s [−1.8 to 0.5 s], Cohen's d = 0.34) compared to a negligible change following waitlist-control (0.2 ± 1.8 s [−1.3 to 1.6 s], Cohen's d = 0.09). However, there was a large lower body strength improvement as represented by a greater number of reps achieved during the 30 s STS test following waitlist-control (+1 ± 3 reps, Cohen's d = 0.78) relative to a negligible change following VR-E (0 ± 2 reps, Cohen's d < 0.01).

## 4 Discussion

This is the first pilot study to evaluate the impact of a work-integrated VR-E program on CHI in individuals with developmental disability. The main finding was that our VR-E program had a trend towards a small improvement in CHI, whereas no intervention or waitlist-control had a negligible influence. Moreover, the present pilot study also showed that VR-E may induce a noticeable improvement in upper body strength and mobility relative to either a small or negligible change following no intervention or waitlist-control. Indeed, this pilot study provides the first glimpse of the importance of a work-integrated VR-E program on CHI and functional capacity in individuals with developmental disability.

This pilot study builds upon the growing body of evidence supporting the benefits of virtual reality-based exercise for individuals with developmental disabilities (Lotan et al., 2010; Li et al., 2023). Similar to traditional exercise interventions (Kastanias et al., 2015; Melo et al., 2024), our VR-E program showed potential for improving cardiovascular health parameters in this population. Notably, the present study is the first to demonstrate that a work-integrated VR-E program may generate sufficient aerobic exercise intensity to promote CHI improvements in people with developmental disability. This is important because an unfavourable CHI as reported in people with developmental disability (Hilgenkamp et al., 2019) have been shown to be independent predictors of CVE (Chirinos et al., 2012) and all-cause mortality (Li et al., 2019). It has been proposed that the CHI improvement with exercise may be due to enhanced arterial compliance (Tanaka and Safar, 2005) and reduced peripheral vascular resistance (Edwards et al., 2004), both leading to better ventricular-vascular coupling (Chantler et al., 2008).

Exercise is known to promote an increase in blood flow and shear stress that acts as a mechanical stimulus to the activation of endothelial nitric oxide synthase resulting in an increase in nitric oxide bioavailability (Michel and Vanhoutte, 2010). This in turn leads to improved arterial compliance and reduced peripheral vascular resistance, and ultimately better ventricular-vascular coupling as reflected by the favourable change in CHI following our VR-E program. Whereas a sedentary or inactive behaviour, as seen in our waitlist-control group, has been shown to result in chronic low shear stress. This increases susceptibility to a heightened presence of biomarkers such as proinflammatory factors (Vion et al., 2013), oxidative stress (Mohan et al., 2007), cell adhesion molecules (Wang and Liao, 2004), and reduced antioxidant expression (Inoue et al., 1996). These factors are associated with vascular dysfunction and ultimately impaired ventricular-vascular coupling as reflected by a negligible change in CHI following an eight-week period in our waitlist-control group.

Moreover, similar to the present study, a systematic review including 13 studies also provided preliminary evidence to demonstrate that participation in VR-E is suitable for individuals with developmental disability and could promote physical fitness improvement in comparison to a control group (Li et al., 2023). Specifically, previous randomised controlled trials (Silva et al., 2017; Hsu, 2016) have also shown a tendency towards a greater mobility improvement, as reflected by the TUG assessment, relative to control or no treatment. An improvement in TUG score is important as it has been demonstrated to be associated with a lower risk of functional dependency occurrence (Lee et al., 2020) and future incidence of cardiovascular disease and mortality (Son et al., 2020). The present study and others (Silva et al., 2017; Hsu, 2016) have also demonstrated that VR-E may promote more muscular strength improvement in comparison to no treatment or control. In particular, our study suggests that VR-E is sufficient to improve upper body strength in employees of a disability employment service (Bedford Group) which involves work activities such as packaging of company products.

Our study findings also align with studies demonstrating the feasibility and potential of VR-E to enhance motivation and engagement towards regular exercise in this population (Li et al., 2023; Barbour et al., 2024). The present study showed a high engagement (91.7 % adherence to the prescribed VR-E sessions) and reports no adverse impacts arising from this type of intervention in this vulnerable population. The novelty of our study lies in the integration of a VR-E program within a workplace setting for individuals with developmental disability, addressing a gap in the current literature. Our study suggests that a workplace-integrated VR-E intervention can promote the attainment of the current exercise guidelines for good cardiovascular health (at least 150 min a week of moderate intensity exercise) (Liguori and Medicine ACoS., 2020), with the average self-selected VR-E intensity reported to be at ‘RPE of 4’ that equates to an intensity above ‘moderate intensity’ (somewhat hard) (Williams, 2017).

### 4.1 Limitations

A limitation of the present pilot study is the small sample size which could explain the absence of a statistical difference between groups. The sample size was merely based on the number of participants from Bedford group who agreed to be included during the recruitment period, forming study groups that is lower than the proposed pilot study sample size flat rule of thumb of 12–35 per group (Kunselman, 2024). Similarly, the VR-E program may not have provided sufficient exercise intensity to enable significant changes in all outcome measures relative to waitlist-control (between-group difference), despite a reported average self-selected VR-E intensity above ‘moderate intensity’ indicated by a validated subjective scale (Borg RPE scale). A larger randomised controlled trial is warranted to confirm our findings. Nevertheless, our pilot study indeed provides the first preliminary evidence towards the cardiovascular health and functional capacity benefits of a workplace-integrated program in people with developmental disability. Another limitation is the mere use of an indirect measure of CHI via the cuff oscillometric method for pulse wave analysis using the cuff-based SphygmoCor Xcel device. However, it should be noted that measurements from this methodology have been demonstrated to have strong correlation with invasive measurements (Shoji et al., 2017). This non-invasive methodology also provides a more practical approach for daily clinical use, with its easy-to-use, operator independent procedure (Shoji et al., 2017).

## 5 Conclusion

Our pilot study provides preliminary evidence to suggest that a workplace-integrated VR-E program may be a safe, motivating, and feasible intervention to promote cardiovascular health (CHI improvement) and functional independence which could potentially translate into improved workplace and productivity longevity in people with developmental disability. A future definitive trial is warranted to confirm the importance of integrating a similar VR-E program across different disability employment services and its impact on overall work productivity and workplace absenteeism in people with developmental disability.

Conflict of interest

Administrative support was provided by Bedford Group. The authors report no relationships that could be construed as a conflict of interest**.**

## CRediT authorship contribution statement

**Joyce S. Ramos:** Writing – review & editing, Writing – original draft, Visualization, Validation, Supervision, Software, Resources, Project administration, Methodology, Investigation, Funding acquisition, Formal analysis, Data curation, Conceptualization. **June Alexander:** Writing – review & editing, Supervision, Project administration, Methodology, Funding acquisition. **Lance C. Dalleck:** Writing – review & editing. **Claire Drummond:** Writing – review & editing. **Alline Beleigoli:** Writing – review & editing. **Belinda Lange:** Writing – review & editing, Methodology. **Caroline Ellison:** Writing – review & editing, Funding acquisition. **Cardiometabolic, Neuro Rehabilitation Group:** Writing – review & editing, Formal analysis.

## Patient consent

Written and oral consent were obtained by an experienced researcher in the disability field prior to inclusion.

## Declaration of competing interest

The authors declare that they have no known competing financial interests or personal relationships that could have appeared to influence the work reported in this paper.

## Data Availability

The data that support the findings of this study are available on request from the corresponding author. The data are not publicly available due to privacy or ethical restrictions.

## Appendix A Supplementary data

Supplementary material 1 Image 1

## References

[bb0005] Barbour B., Sefton L., Bruce R.M., Valmaggia L., Runswick O.R. (2024). Acute psychological and physiological benefits of exercising with virtual reality. PLoS One.

[bb0010] Carmeli E., Imam B. (2014). Health promotion and disease prevention strategies in older adults with intellectual and developmental disabilities. Front. Public Health.

[bb0015] Chantler P.D., Lakatta E.G., Najjar S.S. (2008). Arterial-ventricular coupling: mechanistic insights into cardiovascular performance at rest and during exercise. J. Appl. Physiol..

[bb0020] Chirinos J.A., Kips J.G., Jacobs D.R. (2012). Arterial wave reflections and incident cardiovascular events and heart failure: MESA (multiethnic study of atherosclerosis). J. Am. Coll. Cardiol..

[bb0025] Climie R.E., Schultz M.G., Nikolic S.B., Ahuja K.D., Fell J.W., Sharman J.E. (2012). Validity and reliability of central blood pressure estimated by upper arm oscillometric cuff pressure. Am. J. Hypertens..

[bb0030] Coombes J.S., Skinner T. (2020).

[bb0035] DuBose J. (2022). Discontinued virtual reality systems: the future becoming the past. Public Serv. Q..

[bb0040] Edwards D.G., Schofield R.S., Magyari P.M., Nichols W.W., Braith R.W. (2004). Effect of exercise training on central aortic pressure wave reflection in coronary artery disease. Am. J. Hypertens..

[bb0045] Erickson S.R., Spoutz P., Dorsch M., Bleske B. (2016). Cardiovascular risk and treatment for adults with intellectual or developmental disabilities. Int. J. Cardiol..

[bb0050] Gallego G., Dew A., Lincoln M. (2017). Access to therapy services for people with disability in rural a ustralia: a carers’ perspective. Health Soc. Care Community.

[bb0055] Government A. (2025). Body mass index (BMI) and waist measurement. https://www.health.gov.au/topics/overweight-and-obesity/bmi-and-waist.

[bb0060] Hametner B., Wassertheurer S., Hughes A.D., Parker K.H., Weber T., Eber B. (2014). Reservoir and excess pressures predict cardiovascular events in high-risk patients. Int. J. Cardiol..

[bb0065] Hilgenkamp T.I.M., Schroeder E.C., Wee S.O. (2019). Altered central hemodynamics in individuals with down syndrome. Artery Res..

[bb0070] Hsu T.-Y. (2016). Effects of Wii fit® balance game training on the balance ability of students with intellectual disabilities. J. Phys. Ther. Sci..

[bb0075] Inoue N., Ramasamy S., Fukai T., Nerem R.M., Harrison D.G. (1996). Shear stress modulates expression of cu/Zn superoxide dismutase in human aortic endothelial cells. Circ. Res..

[bb0080] Jacob U.S., Pillay J., Johnson E., Omoya O., Adedokun A.P. (2023). A systematic review of physical activity: benefits and needs for maintenance of quality of life among adults with intellectual disability. Front. Sports Active Living.

[bb0085] Kastanias T.V., Douda H.T., Batsiou S.A., Tokmakidis S.P. (2015). Effects of aerobic exercise on health-related indicators in individuals with intellectual disability with or without the down syndrome. PANR J..

[bb0090] Krahn G.L., Hammond L., Turner A. (2006). A cascade of disparities: health and health care access for people with intellectual disabilities. Ment. Retard. Dev. Disabil. Res. Rev..

[bb0095] Kunselman A.R. (2024). A brief overview of pilot studies and their sample size justification. Fertil. Steril..

[bb0100] Lee J.E., Chun H., Kim Y.-S. (2020). Association between timed up and go test and subsequent functional dependency. J. Korean Med. Sci..

[bb0105] Li W.-f., Huang Y.-q., Feng Y.-q. (2019). Association between central haemodynamics and risk of all-cause mortality and cardiovascular disease: a systematic review and meta-analysis. J. Hum. Hypertens..

[bb0110] Li X., Huang J., Kong Z., Sun F., Sit C.H., Li C. (2023). Effects of virtual reality-based exercise on physical fitness in people with intellectual disability: a systematic review of randomized controlled trials. Games Health J..

[bb0115] Liguori G., Medicine ACoS. (2020).

[bb0120] Lotan M., Yalon-Chamovitz S., Weiss P.L.T. (2010). Virtual reality as means to improve physical fitness of individuals at a severe level of intellectual and developmental disability. Res. Dev. Disabil..

[bb0125] MacDonald C., Bush P.L., Foley J.T. (2022). Physical activity promotion and adults with intellectual disabilities: a neglected area. J. Intellect. Disabil..

[bb0130] Marlow N., Hastings K., Hansson J. (2014).

[bb0135] Melo X., Simão B., Catela C. (2024). Home-vs gym-based exercise delivery modes of two multicomponent intensity training regimes on cardiorespiratory fitness and arterial stiffness in adults with intellectual and developmental disability during the COVID-19 pandemic–a randomized controlled trial. J. Intellect. Disabil..

[bb0140] Michel T., Vanhoutte P.M. (2010). Cellular signaling and NO production. Pflügers archiv-Eur. J. Physiol..

[bb0145] Mohan S., Koyoma K., Thangasamy A., Nakano H., Glickman R.D., Mohan N. (2007). Low shear stress preferentially enhances IKK activity through selective sources of ROS for persistent activation of NF-κB in endothelial cells. Am. J. Phys. Cell Phys..

[bb0150] Pappano A., Wier W. (2013).

[bb0155] Ramos J.S., Ramos M.V., Dalleck L.C. (2016). Fitness is independently associated with central hemodynamics in metabolic syndrome. Med. Sci. Sports Exerc..

[bb0160] Rozak K., Foley J.T., MacDonald C., Bryan R., Lloyd M., Temple V. (2017). Physical activity frequency of Special Olympics athletes aged 8–18 across economic status. Eur. J. Adapted Phys. Activity..

[bb0165] Shoji T., Nakagomi A., Okada S., Ohno Y., Kobayashi Y. (2017). Invasive validation of a novel brachial cuff-based oscillometric device (SphygmoCor XCEL) for measuring central blood pressure. J. Hypertens..

[bb0170] Silva V., Campos C., Sá A. (2017). Wii-based exercise program to improve physical fitness, motor proficiency and functional mobility in adults with down syndrome. J. Intellect. Disabil. Res..

[bb0175] Son K.Y., Shin D.W., Lee J.E., Kim S.H., Yun J.M., Cho B. (2020). Association of timed up and go test outcomes with future incidence of cardiovascular disease and mortality in adults aged 66 years: Korean national representative longitudinal study over 5.7 years. BMC Geriatr..

[bb0180] Tanaka H., Safar M.E. (2005). Influence of lifestyle modification on arterial stiffness and wave reflections. Am. J. Hypertens..

[bb0185] Vacha-Haase T., Thompson B. (2004). How to estimate and interpret various effect sizes. J. Couns. Psychol..

[bb0190] Vázquez A., Jenaro C., Flores N., Bagnato M.J., Pérez M.C., Cruz M. (2018). E-health interventions for adult and aging population with intellectual disability: a review. Front. Psychol..

[bb0195] Vion A.-C., Ramkhelawon B., Loyer X. (2013). Shear stress regulates endothelial microparticle release. Circ. Res..

[bb0200] Wang J.-S., Liao C.-H. (2004). Moderate-intensity exercise suppresses platelet activation and polymorphonuclear leukocyte interaction with surface-adherent platelets under shear flow in men. Thromb. Haemost..

[bb0205] Wassertheurer S., Kropf J., Weber T. (2010). A new oscillometric method for pulse wave analysis: comparison with a common tonometric method. J. Hum. Hypertens..

[bb0210] Williams N. (2017). The Borg rating of perceived exertion (RPE) scale. Occup. Med..

[bb0215] Zablotsky B., Black L.I., Maenner M.J. (2019). Prevalence and trends of developmental disabilities among children in the United States: 2009–2017. Pediatrics.

